# Creative Aging Collaborations: New Directions in Solving Critical Societal Aging and Health Problems

**DOI:** 10.1093/geroni/igaa057.2211

**Published:** 2020-12-16

**Authors:** Jamie Dunlap, Steven Horner, Catherine Richmond-Cullen

**Affiliations:** 1 Pennsylvania Council on the Arts, Harrisburg, Pennsylvania, United States; 2 Pennsylvania Department of Aging, Harrisburg, Pennsylvania, United States; 3 NeuroLEARN, LLC, Moosic, Pennsylvania, United States

## Abstract

From the grassroots development of creative aging research to broad base community collaborations that address the issues of today and tomorrow, this presentation will illustrate how state departments of aging and state arts agencies can combine to partnership with research universities to demonstrate the efficacy of the arts to mediate problems of loneliness, isolation and caregiver stress. This case study will show how resources can be expanded and effective practices established through community based research to find ways to build healthy and engaging communities that serve to break down the barriers of isolation and promote social networks.

